# The Impact of Uremic Toxins on Cerebrovascular and Cognitive Disorders

**DOI:** 10.3390/toxins10070303

**Published:** 2018-07-22

**Authors:** Maryam Assem, Mathilde Lando, Maria Grissi, Saïd Kamel, Ziad A. Massy, Jean-Marc Chillon, Lucie Hénaut

**Affiliations:** 1 Nephrology Department, Amiens University Hospital, 80054 Amiens, France; maryam_assem@hotmail.com (M.A.); mathilde.lando@hotmail.fr (M.L.); 2 MP3CV, EA7517, CURS, Jules Verne University of Picardie, 80054 Amiens, France; grissimaria@gmail.com (M.G.); said.kamel@u-picardie.fr (S.K.); lucie.henaut@gmail.com (L.H.); 3 Biochemistry Laboratory, Amiens University Hospital, 80054 Amiens, France; 4 Division of Nephrology, Ambroise Paré University Hospital, APHP, Boulogne-Billancourt, 92100 Paris, France; ziad.massy@apr.aphp.fr; 5 Inserm U1018, Team 5, CESP, UVSQ, Paris-Saclay University, 94800 Villejuif, France; 6 DRCI, Amiens University Hospital, 80054 Amiens, France

**Keywords:** stroke, dementia, cognitive disorders, uremic toxins

## Abstract

Individuals at all stages of chronic kidney disease (CKD) have a higher risk of developing cognitive disorders and dementia. Stroke is also highly prevalent in this population and is associated with a higher risk of neurological deterioration, in-hospital mortality, and poor functional outcomes. Evidence from in vitro studies and in vivo animal experiments suggests that accumulation of uremic toxins may contribute to the pathogenesis of stroke and amplify vascular damage, leading to cognitive disorders and dementia. This review summarizes current evidence on the mechanisms by which uremic toxins may favour the occurrence of cerebrovascular diseases and neurological complications in CKD.

## 1. Introduction

Chronic kidney disease (CKD) is associated with alterations of vascular functions, blood chemistry, and red blood cell production [1]. These dysfunctions impair blood perfusion, and this can subsequently affect brain functions. Indeed, cerebrovascular diseases are common in CKD patients, who display an increased rate of cognitive disorders and dementia [2,3] and a greater burden of abnormal brain white matter disease [4]. Cognitive impairments in CKD can be partly related to vascular dementia, as dialysis patients with cognitive disorders also present numerous cortical ischemic lesions [5,6,7]. Indeed, the risk of transient ischemic attack and stroke increases with progressive kidney function decline [8,9,10,11,12] and stroke in these patients is associated with more severe neurological deterioration, poorer functional recovery [13,14], and increased mortality [15,16].

In addition to traditional cardiovascular risk factors, such as hypertension, diabetes, inflammation, and dyslipidemia, non-traditional risk factors related to kidney injury may predispose CKD patients to cerebrovascular diseases. These non-traditional risk factors include the CKD-associated disorders of bone and mineral metabolism (CKD-MBD), inflammation, and oxidative stress. Oxidative stress is a key inducer of peripheral vascular dysfunction, characterized by impaired endothelial function, increased atherosclerosis, and vascular calcification. These vascular dysfunctions, which alter the general hemodynamics, increase the risk of cerebrovascular events, such as stroke. In addition, reactive oxygen species (ROS) exert damaging effects on the cerebral vasculature during stroke by increasing vasodilation, altering vascular reactivity, and destroying the blood-brain barrier (BBB) [17,18]. Oxidative entities also contribute to post-stroke DNA damage and cell death during ischemic stroke [19]. In patients with neurodegenerative diseases, ROS production exacerbates the expression of inflammatory mediators [20]. This neuroinflammation, together with the increased oxidative stress, contributes to the pathogenesis of neuronal degeneration [21] and can cause cell membrane damage as a result of lipid peroxidation, changes in protein structure and function due to protein oxidation, and structural DNA damage, hallmarks of several neurodegenerative diseases [22,23].

**Figure 1:**
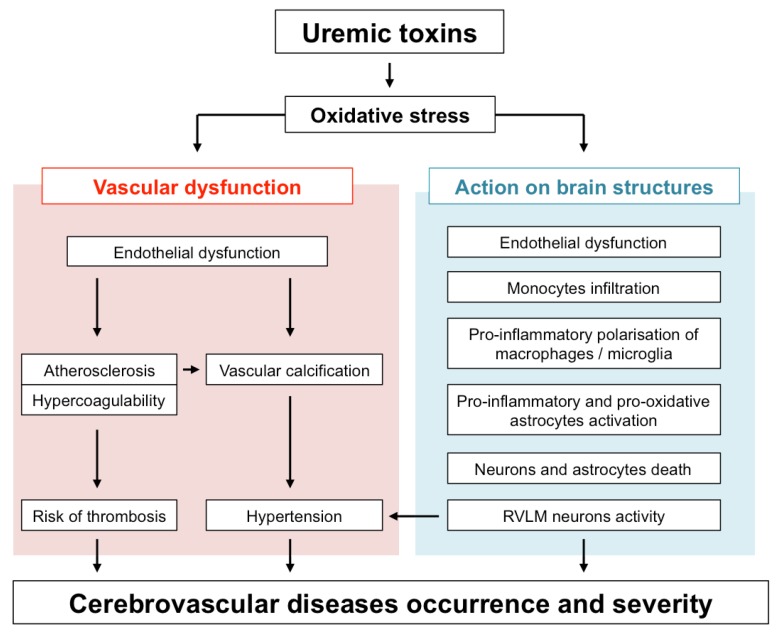
Impact of uremic toxins-induced oxidative stress on the occurrence and severity of cerebrovascular diseases. RVLM: rostral ventrolateral medulla.

Protein-bound uremic toxins (UTs) such as indoxyl sulfate (IS) or paracresyl sulfate (PCS) are strong inducers of oxidative stress (for review see [24]). IS, PCS and guanidino compounds are highly expressed in uremic brains [25,26]. In addition, cerebrospinal fluid and brain levels of certain guanidine compounds, such as creatinine and methylguanidine, are substantially elevated in uremic patients [26]. Interestingly, these high toxin concentrations (up to 10-fold higher in CKD patients than in controls) were found in brain regions that play a determinant role in cognition, such as the thalamus, mammillary bodies, and cerebral cortex [26]. In this context, it is tempting to speculate that UTs-induced oxidative stress in these brain regions may directly impact the local microcirculation as well as brain-resident cells, therefore contributing to cognitive disorders and poor stroke recovery in CKD patients. This review summarizes our current knowledge on the mechanisms by which UTs-induced oxidative stress may promote both large vessels and microvascular dysfunction and how their subsequent effect on infiltrated macrophages, microglia, astrocytes, and neurons promotes brain damage. An overview of these mechanisms is provided in Figure 1.

## 2. Impact of Uremic Toxins on Large Vessels Functionality

### 2.1. Regulation of Blood Pressure

#### 2.1.1. IS, Uric Acid, and Methylguanidine

Hypertension is the main risk factor for stroke [27]. Patients with CKD often present hypertension, but the cause of this hypertension, apart from sodium and fluid retention, has not been fully elucidated. The rostral ventrolateral medulla (RVLM), which contains presympathetic neurons, is known to be a pivotal region that regulates blood pressure [28]. According to a recent study, superfusion of bulbospinal RVLM neurons with uric acid, IS, and methylguanidine increased their activity, as evidenced by depolarization and an increased number of action potentials [29]. In this study, UT-induced activities of the RVLM neurons were suppressed by the addition of an antioxidant drug, suggesting that UT-induced oxidative stress plays a key role in bulbospinal RVLM neuron activation. The authors concluded that ROS production by UTs may cause hypertension by activating RVLM neurons [29]. Since antihypertensive drugs may decrease age-related cognitive decline and dementia [30,31], and given the positive correlation observed between hemodynamic impairment and cognitive impairment in the early stages of dementia [32,33], targeting UT-induced activation of RVLM neurons could be a promising approach to reduce hypertension and subsequent neurological complications.

#### 2.1.2. Lanthionine

Uremic patients undergoing hemodialysis display high concentrations of the sulfur amino acid derivative lanthionine, which has been recently proposed as a UT [34]. Lanthionine, a natural nonproteogenic amino acid, is an analog of cysteine [35], which is considered as a stable product of hydrogen sulphide (H_2_S) metabolism and an indirect biomarker of its production [36,37]. Cystathionine β-synthase (CBS) and cystathionine γ-lyase (CSE), two key enzymes in H_2_S production, utilize as substrates cysteine and homocysteine, whose levels are both increased in uremic patients [36]. Interestingly, lanthionine is involved in CBS inhibition [38], which impairs H_2_S production in cell cultures [34].

In recent years, H_2_S, which is considered as the third gaseous mediator with nitric oxide (NO) and carbon monoxide (CO), has become recognized as an important endogenous vasodilator [39,40,41]. In the first report on this subject, H_2_S relaxed rat aortic tissues in vitro [42]. In this study, exposure to H_2_S enhanced NO-induced smooth muscle relaxation in the thoracic aorta, suggesting that the endogenous H_2_S may regulate smooth muscle tone in synergy with NO. In a subsequent study, an intravenous bolus injection of H_2_S was shown to transiently decrease rats’ blood pressure [43]. In this study, a small portion of the H_2_S-induced vasorelaxation was attenuated by either removal of the endothelium or the application of L-NAME (an inhibitor of NO synthase) in the presence of the endothelium, suggesting that a part of the vasodilator actions of H_2_S results from NO production. Confirming these data, mice displaying decreased H_2_S production due to genetic deletion of CSE showed significant hypertension and diminished endothelial vasorelaxation [44]. In this context, it is conceivable that the hypertension observed in CKD may be linked, at least in part, to lanthionine-induced CBS inhibition and subsequently impaired H_2_S production. Further studies will be needed to clarify this concern.

### 2.2. Vascular Dysfunction

Evidence from in vitro and clinical studies suggests that the actions of UTs on the vascular system may predispose to neurological disorders. For instance, UT-induced oxidative stress accelerates the progression of atherosclerosis [45,46] and endothelial dysfunction [47,48,49,50,51], both of which are associated with an increased risk of dementia and stroke [52,53]. In addition, inorganic phosphate (Pi), IS, TNF-α, IL-6, and advanced glycation end-products have been reported to promote the development of vascular calcification [54,55], at least in part through increased oxidative stress. This phenomenon rigidifies arteries and contributes to the onset of hypertension, the main risk factor for stroke. Of interest, the presence of intracranial artery calcification has been reported to be associated with mortality and vascular events after hospital discharge in patients with ischemic stroke [56]. Increased intracranial artery calcification in response to UTs in CKD could, therefore, be responsible for the poor stroke outcomes observed in CKD patients.

### 2.3. Hemostasis

Thrombosis and hemostasis impact both the occurrence and outcomes of cerebrovascular disease [57]. Of interest, patients with end-stage renal disease (ESRD) exhibit features of a hypercoagulable state [58]. Normal endothelium exerts anticoagulant and antithrombotic effects and, therefore, plays a pivotal role in hemostasis [59]. Indeed, platelet adhesion and aggregation are inhibited by glycosaminoglycan, NO, prostacyclin, endothelin-1, and ectonucleotidases present in endothelial cells. Interestingly, UTs such as IS have been reported to be associated with enhanced hemostatic disorders and may, therefore, represent interesting therapeutic targets in the prevention of cerebrovascular disease.

#### 2.3.1. Indoxyl Sulfate

In 2011, Kaminski and colleagues reported the existence of a correlation between hemostatic factors such as tissue factor, von Willebrand factor, thrombomodulin, soluble urokinase-type plasminogen activator receptor, soluble intercellular adhesion molecule-1, and soluble vascular cell adhesion protein and the fraction of IS in CKD patients not undergoing hemodialysis [60]. In this study, levels of IS were independently associated with markers of impaired endothelial function (thrombomodulin, adhesion molecules), oxidative stress (superoxide-dismutase), and monocyte activation (neopterin). Interestingly, parameters that correlated with the levels of IS (von Willebrand factor, soluble urokinase-type plasminogen activator receptor, soluble intercellular adhesion molecule-1) were positively associated with the prevalence of cardiovascular disease in CKD patients. The authors concluded that IS may be one of the key links existing between impaired renal function and the prevalence of cardiovascular events, especially hemostatic disorders, through altered monocyte activation, intensified inflammatory processes, and augmented oxidative stress. 

#### 2.3.2. Homocysteine

Similarly, hyperhomocysteinemia, which is common in CKD patients [61], has a direct prothrombotic effect on the vascular system and may therefore lead to both large- and small-vessel disease [62]. Homocysteine is a thiol-containing amino acid derived from the metabolism of dietary methionine. Moderately elevated plasma homocysteine levels are an important independent risk factor for arterial and venous thrombosis [63,64]. Numerous mechanisms have been postulated by which hyperhomocysteinemia may induce thrombosis. Indeed, early studies performed in rabbits showed that hyperhomocysteinemia is associated with abnormalities in the key coagulation protein fibrinogen [65,66]. This acquired dysfibrinogenemia is characterized by formation of clots composed of abnormally thin, tightly packed fibers with an increased resistance to fibrinolysis. These data suggested that hyperhomocysteinemia might directly promote thrombosis by interfering with the normal process by which intravascular clots are removed [67]. Most hypotheses also involve injury to the vascular endothelium [68,69] or some alteration in endothelial function, such as decreased expression of the anticoagulant regulatory protein thrombomodulin [70], anticoagulant heparans [71], or binding sites for tissue plasminogen activator [72,73]. In addition, data from in vitro studies reported that the induction of tissue factor expression by cultured umbilical vein endothelial cells [74] and circulating monocytes might be a plausible mechanism by which homocysteine may induce thrombosis [75]. Deficiency of folic acid is a treatable cause of hyperhomocysteinemia. Interestingly, the decrease in homocysteine observed after folic acid treatment in human is accompanied by a decrease in the procoagulatory potential, characterized by decreased fibrinogen (with a procoagulation potential) and increased plasminogen (with an anti-coagulatory potential) [76]. Confirming the potential role of homocysteine-induced thrombosis on the onset of cerebrovascular disease, the China Stroke Primary Prevention Trial recently showed the benefits of folic acid, as a mean to reduce homocysteinemia and to prevent stroke in Chinese adults with hypertension [77].

#### 2.3.3. Other UTs

Guanidino compounds, that are known to be associated with cognitive disorders as discussed below, have been shown to induce elevation of serum homocysteine [78]. In the same manner, CBS inhibition by lanthionine might be responsible for the degree of hyperhomocysteinemia observed in uremia. This suggests that these UTs may also impair brain function indirectly through dysregulated hemostasis. Therefore, targeting lanthionine and guanidino compounds may represent a promising strategy to reduce the hyperhomocysteinema and subsequent dysregulated hemostasis observed in CKD. The hypercoagulability observed in ESRD patients could also be linked to the kynurenine (KYN) pathway since KYN metabolites were reported to be significantly associated with elevated prothrombin factors 1 + 2 in dialysis patients [58].

### 2.4. Atrial Fibrillation

CKD is associated with a higher incidence of atrial fibrillation [79], which increases the risk of thromboembolic stroke, heart failure, and mortality [80]. However, the arrhythmogenic mechanisms linked to CKD are not fully elucidated.

#### Indoxyl Sulfate

In ex vivo experiments, exposure to IS increased rabbits pulmonary vein and left atrium arrhythmogenesis and reduced sinoatrial nodes pacemaker activity through oxidative stress [81]. In addition, data obtained from in vitro experiments reported that exposure to IS significantly increased neonatal rat cardiac fibroblast collagen synthesis and myocyte hypertrophy [82]. In this study, IS also stimulated TNF-α, IL-6, and IL-1β mRNA expression in THP-1 cells, which demonstrated, for the first time, that IS has pro-fibrotic, pro-hypertrophic, and pro-inflammatory effects and might, therefore, play an important role in adverse cardiac remodeling. These effects of IS on cardiac remodeling and cardiac electrophysiology, together with its previously discussed thrombogenic properties, may contribute to the higher prevalence rate of atrial fibrillations and subsequent stroke occurrence in CKD patients. Further studies will be needed to clarify this concern.

## 3. Impact of Uremic Toxins on Brain Microcirculation

### 3.1. Endothelial Cells

Over recent years, the major impact of small vessel disease (SVD) on cognitive impairment has been clearly recognized. SVD is a systemic disease, probably related to diffuse endothelial dysfunction, which affects the perforating arterioles, capillaries, and venules in the brain. Cerebral SVD causes focal lacunar infarction and more diffuse ischemia, referred to as leukoaraiosis [83]. Although often asymptomatic, it is responsible for almost half of all cases of dementia and for a significant proportion of stroke cases [84].

#### 3.1.1. Uremic Toxins and Vasoreactivity

##### Phosphate and Indoxyl Sulfate

As discussed below, UTs are strong inducers of large vessel endothelial dysfunction. In addition, patients with renal disease are also prone to peripheral microvascular dysfunction [85]. Therefore, in 2011, our group evaluated the impact of uremia on the function of the cerebral microcirculation in a mouse model [86]. In this study, we demonstrated that the endothelium-dependent relaxation of cerebral microvessels was impaired during CKD while the endothelium-independent relaxation was not. In addition, we observed that plasma concentration of asymmetric dimethyl arginine (ADMA), an UT and endogenous inhibitor of eNOS, was elevated in CKD mice as compared to SHAM-operated mice, which suggested that these alterations were related, at least in part, to a decrease in NO production. In line with this hypothesis, we subsequently reported that the in vitro exposure to IS or high-Pi induced cerebral endothelial cells dysfunction by decreasing NO levels due to enhanced oxidative stress [87]. Interestingly, infusion of ADMA in 10 healthy volunteers was reported to decrease cerebral perfusion and arterial compliance [88]. Altogether, these data suggest that UTs may induce endothelial dysfunction not only in large vessels, but also in cerebral microvessels via increased ROS production, a phenomenon that may directly promote SVD and, subsequently, stroke and cognition. In line with these data, spatial working memory was reported to be impaired in mice subjected to eight weeks of CKD, which is known to exhibit increased circulating levels of UTs and increased oxidative DNA damage [89]. However, there is no direct evidence of a potential link between UT-induced ROS production, SVD, and subsequent cognitive impairment or stroke at the present time.

##### Homocysteine

Elevated homocysteine levels are observed in 85% of dialysis patients, but only in 10% of the general population [61]. In a prospective cohort study, plasma homocysteine was reported to be an independent risk factor for dementia [90]. In addition, elevated plasma homocysteine concentrations are associated with an increased risk for Alzheimer’s disease [91]. Furthermore, recent cross-sectional data provide evidence that a higher level of total homocysteine is associated with white matter hyper-intensity volume, suggesting that total homocysteine is a risk factor for white matter damage [92]. Interestingly, data obtained from in vitro [69,93] and in vivo studies [94,95] suggest that the effect of homocysteine on the number and progression of white matter lesions might be mediated through direct endothelial damage [6]. Indeed, in vitro exposure of endothelial cells to homocysteine had been reported to reduce cell adherence and to decrease the bioavailability of endothelium-derived NO [69,93]. In line with these data endothelium-dependent vasodilation, measured with high-resolution ultrasonography, was reported to be significantly impaired in hyperhomocysteinemic subjects compared with control subjects both in middle-aged [95] and elderly people [94], suggesting that the bioavailability of NO is decreased in hyperhomocysteinemic humans. Therefore, in 2004, Hassan and colleagues intended to determine whether elevated homocysteine levels is a risk factor for SVD and whether this association was mediated by endothelial dysfunction as assessed by circulating endothelial markers [83]. In their study, performed on 172 Caucasian patients with SVD and 172 community controls of similar age and sex, mean homocysteine levels were higher in SVD patients than in control subjects and homocysteine was a stronger risk factor in those with ischemic leukoaraiosis in comparison with isolated lacunar infarction. In addition, homocysteine levels were associated with the markers of endothelial dysfunction intercellular adhesion molecule 1 (ICAM-1) and thrombomodulin. Inclusion of these markers as covariates reduced the association with homocysteine, but improved the overall logistic regression model for prediction of SVD. These findings are consistent with the hypothesis that endothelial dysfunction is an important mechanism through which homocysteine mediates its effects in SVD, particularly in ischemic leukoaraiosis. Together, these data suggest that homocysteine-lowering therapy may be promising to reduce SVD in patients with CKD.

#### 3.1.2. Uremic Toxins and Endothelial Cell Integrity

##### Phosphate, Indoxyl Sulfate, Oxalic Acid, and Homocysteine

UTs have been reported to directly alter the integrity of both large- and small-vessels endothelial cells. For instance, Pi increases the expression of adhesion molecules, such as vascular cell adhesion molecule 1 (VCAM-1) and ICAM-1 [96], two inflammation markers involved in leukocyte adhesion and rolling on endothelial cells. Phosphate may, therefore, predispose to local cerebral inflammation via increased VCAM-1 and ICAM-1 expression in cerebral endothelial cells, thereby contributing to neuroinflammatory diseases in CKD patients. Similarly, administration of IS to normal rats [97] or nephrectomized mice [98] induces leukocyte adhesion to the vascular wall. IS has also been reported to promote senescence of large-vessel endothelial cells via activation of p53 and ROS production [99]. Interestingly, IS also generates disruption of contact between pulmonary artery endothelial cells via phosphorylation of myosin light chain kinase (MLCK) and myosin light chains (MLC) and activation of ERK1/ERK2 [100]. Exposure to uremic levels of oxalic acid for a few days inhibits replication and migration of large-vessel endothelial cells in a concentration- and time-dependent manner [101]. Interestingly, in vitro studies have shown that homocysteine damages endothelial cells by increasing H_2_O_2_ production [102], affecting antioxidant defence systems [103] and triggering apoptosis via mitochondrial oxidant production [104]. Low concentrations of homocysteine were also reported to decrease endothelial cells proliferation [105]. According to several investigators, the toxic effects of homocysteine may be caused, at least in part, by the oxidant species H_2_O_2_, which is generated when homocysteine auto-oxidizes to the disulphide homocysteine or when it forms mixed disulphides with other thiols [69]. Altogether, these data suggest that, by exerting a similar action on both large vessels and microvessels, UTs such as IS, Pi, oxalic acid and homocysteine may predispose to cerebral inflammation by increasing BBB disruption, leukocyte adhesion, rolling and extravasation in brain tissue.

##### Lanthionine

In mice that underwent transient medial cerebral artery occlusion, administration of H_2_S donors decreased the infarction volume and improved neurological deficits [106]. In this model, the beneficial effects of H_2_S donors were related to a reduction of BBB permeability, brain edema and preserved expression of tight junction proteins in the ischemic brain. The authors demonstrated that H_2_S donors protected BBB integrity following experimental stroke possibly by acting through NF-κB inhibition to suppress neuroinflammation induction of MMP9 and NOX4-derived free radicals [106]. Therefore, the possibility that lanthionine-induced impaired H_2_S production may be involved in the poor post-stroke functional outcomes observed in CKD patients through disruption of BBB integrity cannot be ruled out.

#### 3.1.3. Uremic Toxins and Angiogenesis

Angiogenesis is considered as a natural defence mechanism helping to restore oxygen and nutrient supply to the ischemic brain tissue [107]. Therefore, greater microvessels density in the ischemic border correlates with longer survival in stroke patients [108]. As discussed previously, the pathological mechanism of vascular cognitive impairment involves ischemic lesions in the hippocampus. Vascular endothelial growth factor (VEGF) is known to promote angiogenesis and enhances blood flow to ischemic regions. Indeed, induction of VEGF expression in rats with vascular cognitive impairment was reported to increase the number of blood vessels in the hippocampal region and to improve cognitive function and neuronal cell loss [109]. Vascular endothelial growth factor was also reported to promote angiogenesis and functional recovery in rats with stroke [110].

##### Lanthionine

Hydrogen sulphide was reported to stimulate the proliferation and migration of endothelial cells cultured in vitro [111,112]. In these cells, H_2_S also significantly increased tube-like structure formation in in vitro matrigel assays. Interestingly, H_2_S has been shown to induce angiogenesis indirectly through the release of VEGF from hypoxic smooth muscle cells [113]. In line with this observation, exposure of endothelial cells to VEGF increased the production of H_2_S and endogenously produced H_2_S was shown to participate in the angiogenic signalling of VEGF [111]. As a consequence, intraperitoneal administration of the H_2_S donor NaHS in mice increased neovascularization [112]. Therefore, the possibility that lanthionine-induced impaired H_2_S production may be involved in the poor functional post-stroke recovery and cognitive disorders observed in CKD through impaired angiogenesis cannot be ruled out. Further studies will be needed to clarify this concern.

### 3.2. Monocytes/Macrophages

Inflammation is enhanced in CKD patients [114,115,116] and inflammation markers are associated with increased morbidity and mortality in ESRD [117,118]. In dialysis patients, C-reactive protein (CRP) is predictive of stroke and death [119]. Several UTs, such as IS and PCS, exert pro-inflammatory effects and their serum concentrations are correlated with inflammatory markers in CKD patients [116]. Some of these inflammatory markers, such as TNF-α, IL-6, and IL-1β, are currently considered to be UTs [120]. This enhanced state of inflammation appears to play a major role in the neurological complications associated with CKD [24]. A recent review described the various mediators of post-ischemic inflammation [121]. Some of these mediators, such as IL-1α, IL-1β, and TNF-α, are involved in initiation of neuroinflammation, whereas others, including IL-1, 6, 10, 17, 20, and once again TNF-α, contribute to the amplification of neuroinflammation. In contrast, factors such as TGF-β, IL-10, 17, and 23 contribute to the resolution of neuroinflammation [121]. Data from in vitro studies and animal models suggest that the inflammation induced by UTs, such as IS, symmetric dimethylarginine (SDMA), guanidino compounds, or quinolinic acid (QUIN) may contribute to the increased risk of stroke and cognitive impairment observed in CKD patients.

#### 3.2.1. Indoxyl Sulfate

As discussed above, the increased expression of ICAM-1, VCAM-1 and concomitant alteration of endothelial cell interactions observed in response to UTs raise the hypothesis that UTs may promote infiltration of inflammatory monocytes in brain tissue. For example, IS administration in mice has been reported to enhance TNF-α-induced recruitment of leukocytes to the vascular wall [122]. Confirming this observation, data from in vitro studies indicated that IS dose-dependently increased THP-1 monocyte adhesion to IL-1β-activated human endothelial cells under physiological flow conditions [123]. Leukocyte adhesion to the inflamed endothelium involves the β2-integrin family of receptors (which share a common β2 subunit), such as LFA-1 (CD11a/CD18), Mac-1 (CD11b/CD18), p150,95/CR4 (CD11c/CD18), and CD11d/CD18 [124,125]. Mac-1 is a receptor for ICAM-1 and extracellular matrices, abundantly present in injured tissue. Mac-1 expression and ROS production have been reported to be significantly higher in peripheral blood monocytes of subtotal nephrectomised CKD mice than in sham-operated mice [123]. In this model, treatment with AST-120, an oral adsorbent used in the clinic to reduce plasma IS levels, significantly decreased both Mac-1 expression and ROS production, raising the possibility that IS-induced Mac-1 expression may promote leukocyte recruitment to the vascular wall, thereby promoting inflammation.

Depending on the tissue microenvironment, macrophages can be driven to a classically activated pro-inflammatory phenotype (M1) by stimuli such as interferon-γ (IFN-γ), or an alternatively activated anti-inflammatory phenotype (M2) by factors including IL-4 and IL-13. A recent study reported that uremia increased M1 and impaired M2 polarization of macrophages by inhibition of the adenosine monophosphate (AMP)-activated protein kinase (AMPK) [126]. These data suggest that the UTs that accumulate in brain tissue during CKD might impact the polarization of infiltrated monocytes/macrophages. In line with this observation, IS has been reported to directly induce monocyte-mediated inflammation and ROS production in THP-1 monocytes via the NADPH oxidase and MAPK pathways [123] and display direct pro-inflammatory effects in vitro via activation of the NF-kB and MAPK pathways in macrophages differentiated from THP-1 cells [127]. In addition, in a recent study performed on in vitro THP-1 cell cultures, IS was reported to promote CD163 expression and transition to macrophages with the hallmarks of classical M1 (IL-6, CCL2, COX2) and alternative M2 (IL-10, PPARγ, TGF-β, TIMP-1) phenotypes via AhR/Nrf2 activation. The authors concluded that IS may skew monocyte differentiation toward low-inflammatory, profibrotic macrophages and may therefore contribute to persistence of chronic inflammation [128]. Post-stroke intracerebral monocyte recruitment following BBB disruption largely contributes to increased cerebral inflammation and subsequent aggravation of ischemic lesions. It is, therefore, conceivable that IS may amplify post-stroke brain inflammation by promoting monocyte recruitment and macrophage-induced inflammation after BBB disruption and this hypothesis should be tested in future studies.

#### 3.2.2. Dimethylarginines

High serum levels of dimethylarginines (DMA) (both symmetric and asymmetric) are associated with greater stroke severity and deleterious stroke outcomes [129,130,131]. Of interest, SDMA has been reported to play a role in the inflammatory state observed in CKD by activating NF-κB, thereby increasing the expression of pro-inflammatory cytokines, such as IL-6 and TNF-α. Consequently, SDMA levels are associated with inflammatory markers, such as CRP in CKD patients [132]. Induction of inflammation and leukocyte activation by SDMA contributes to cardiovascular complications in uremic patients. Interestingly, SDMA levels have recently been reported to be associated with mediators of inflammation after acute stroke [133], suggesting a possible association between these compounds, inflammation, and stroke. Further work is needed to elucidate the impact of SDMA-induced inflammation on ischemic stroke lesions and subsequent recovery.

#### 3.2.3. Guanidino Compounds

Guanidino compounds appear to exert dual effects on inflammation, as methylguanidine and guanidinoacetic acid increase TNF-α production in monocytes, while guanidinosuccinic acid exerts an inhibitory effect on TNF-α production in monocytes [134]. Whether these dual effects play a role in modulating neuroinflammatory diseases in CKD needs to be clarified.

#### 3.2.4. Quinolinic Acid

During inflammation, the KYN pathway can be activated by cytokines, particularly IFN-γ, leading to the production of the protein-bound uremic excitotoxin QUIN by monocyte lineage cells [135,136]. Cerebral intravascular infusion of QUIN has been reported to enhance permeability of rat brain microvessels to plasma albumin [137]. The extracellular tissue concentration of albumin was consequently increased in the hippocampus proper and striatum, an effect associated with more severe neuronal loss. A vicious circle may therefore exist, in which cerebral inflammation amplifies cerebral inflammation via increased monocyte-induced QUIN secretion and subsequent microvessel permeability. In line with these data, QUIN neurotoxicity has been shown to be involved in the pathogenesis of several age-related neurodegenerative processes associated with neuroinflammation, including Alzheimer’s disease [138,139]. Indeed, decreased 3-hydroxykynurenine and QUIN concentrations have been observed in models of Huntington’s disease [140] and depression [141]. Schizophrenia and autism have been associated with increased kynurenic acid [142], and increased 3-hydroxykynurenine and decreased KYN and kynurenic acid concentrations have been observed in Parkinson’s disease [143]. In this context, QUIN might represent an important target to cure neuroinflammatory diseases in CKD. Further studies are needed to clarify this issue.

#### 3.2.5. Homocysteine

Exposure of cultured primary human monocytes to homocysteine has been reported to increase protein secretion and mRNA expression, as well as activity of monocyte chemoattractant protein-1 (MCP-1) and IL-8, an effect mediated by ROS through NAD(P)H oxidase [144]. MCP-1 is considered as the strongest monocyte chemoattractant and IL-8 is known to be an important chemotactic factor for neutrophils. Therefore, the hyperhomocysteinemia observed in CKD patients may have a key role in leucocyte trafficking at the BBB.

#### 3.2.6. Lanthionine

Hydrogen sulphide is an important inhibitor of acute inflammation, acting at the leukocyte-endothelium interface. Indeed, the administration of the H_2_S donor NaHS in rats suppressed nonsteroidal anti-inflammatory drug-induced granulocyte infiltration, expression of endothelial and leukocyte adhesion molecules, and expression of TNF-α [145]. Administration of H_2_S donors to rats also inhibited aspirin-induced leukocyte adherence and infiltration as well as carrageenan-induced paw edema [146]. In addition, H_2_S has been reported to induce neutrophil apoptosis, thereby contributing to resolution of inflammatory reactions [147]. In this context, targeting lanthionine, which impairs the production of H_2_S, appears as a promising strategy to reduce both the endothelial dysfunction and the inflammatory processes related to cerebrovascular diseases.

#### 3.2.7. Other UTs

Other UTs may also be involved in stroke severity in CKD patients by increasing pro-inflammatory pathways. This is the case for uric acid that activates pro-inflammatory pathways, especially MAPK signalling in vascular smooth muscle cells [148], phenylacetic acid that promotes inflammation, increasing the risk of cardiovascular disease in the presence of uremia [149], and KYN that are associated with inflammation in CKD patients and which may play a role in cardiovascular disease, including stroke [150]. High circulating levels of β-2-microglobulin (B2M) have also recently been reported to be associated with an increased risk of ischemic stroke in women [151]. Since B2M levels correlate with CRP, TNF-α, IL-6, and cardiovascular risk factors in hemodialysis patients [152], this UT could possibly increase the risk of stroke by acting on inflammation.

## 4. Impact of Uremic Toxins on Brain Resident Cells

Renal impairment is associated with the accumulation of UTs within the cerebrospinal fluid and the brain tissue [25,26]. The accumulation of UTs within brain structures is thought to be linked to the expression of their transporter at the BBB and the blood-cerebrospinal fluid barrier (BCSFB) [153,154,155,156,157,158]. Indeed, recent studies show that IS undergoes efflux transport at the BBB via OAT3 [153,159]. In the same manner, guanidino compounds were reported to undergo efflux transport at the BCSFB via OCT3 [153,157,160]. Evidence obtained from in vivo and in vitro studies suggest that, once infiltrated within brain structures, these UTs may have deleterious impact on brain resident cells such as microglia, astrocytes, and neurons.

### 4.1. Microglia

Circulating macrophages that infiltrate into the inflamed central nervous system exert their effects in combination with resident microglia, the major immune cells present in the brain. Like macrophages, microglia, are highly plastic and can assume different functional phenotypes within brain lesions that critically influence the damage and the tissue repair that occurs following injury [161,162]. It has been clearly established that local microglia and newly-recruited macrophages assume a M2 phenotype at early stages of ischemic stroke, but are gradually transformed into the M1 phenotype in peri-infarct regions [163]. M1-polarized microglia/macrophages exacerbate neuronal death and are deleterious to tissue recovery following injury, while M2-polarized microglia/macrophages protect neurons against ischemia and enhance post-injury tissue repair [161,163,164,165]. Since UTs, such as IS, promote macrophage polarization toward the M1 phenotype, they may also concomitantly promote the polarization of resident microglia toward a pro-inflammatory phenotype. Further studies are needed to clarify this possibility.

#### Kynurenine Pathway

Kynurenine pathway metabolic balance has recently been reported to influence microglia activity [166]. The KYN pathway consists of two functionally distinct branches that generate both neuroactive and oxidatively reactive metabolites. In the brain, the rate-limiting enzyme for one of these branches, kynurenine 3-monooxygenase (KMO), is predominantly expressed in microglia and has emerged as a pivotal point of metabolic regulation [167]. In vitro exposure of murine microglia to lipopolysaccharide (LPS) promoted a dose-dependent increase in mRNA expression of IL-1β, IL-6, TNF-α, and inducible NO synthase (iNOS), together with the rate-limiting enzymes of the oxidative KYN pathway, indoleamine-2,3-dioxygenase (IDO)-1, and KMO [166]. Kynurenine and QUIN levels were increased in the medium 24 hours post-LPS. Inhibition of KMO by Ro 61-8048 following LPS challenge attenuated extracellular nitrite accumulation and LPS-induced expression of KMO and TNF-α. Similarly, primary microglia isolated from KMO-/- mice exhibited a significantly reduced pro-inflammatory response to LPS compared to WT controls. Altogether, these data suggested that targeting KMO may dampen neuroinflammation, which may impact the subsequent neurological disorders observed in CKD.

### 4.2. Astrocytes

In healthy neural tissue, astrocytes play critical roles in energy provision, regulation of blood flow, homeostasis of extracellular fluid, ions and transmitters, regulation of synapse function, and synaptic remodeling. Of interest, astrocytes respond to all forms of central nervous system insults, such as trauma, ischemia, or neurodegenerative diseases, by a process commonly referred to as reactive astrogliosis. Reactive astrogliosis involves changes in astrocyte molecular expression and morphology and, in severe cases, scar formation [168]. Reactive astrogliosis can exert both beneficial and detrimental effects in a context-dependent manner determined by specific molecular signalling cascades.

Astrocyte degeneration has been described in several pathological conditions, such as depressive disorders or dementia [169]. Reactive astrogliosis and dystrophy accompany these diseases and may directly contribute to the early alterations in synaptic transmission and cognitive processes that occur prior to neurodegeneration. In addition, astroglial cell loss described at later stages of several neurodegenerative diseases is also likely to indirectly alter neuronal function and survival by compromising glial physiological support and modulating neuronal activity, and may consequently accelerate the course of the disease [169,170].

#### 4.2.1. Methylguanidines

Dementia is a neurological disease observed more commonly in uremic patients than in the general population and several types of dementia are associated with astroglial apoptosis. In a rat glioma cell line (C6) cultured in vitro, pre-incubation with methylguanidine significantly increased H_2_O_2_-induced cell death [171]. In this study, the fluorescent dye FURA 2-AM test showed significant elevation of [Ca^2+^]_i_ in C6 cells co-treated with methylguanidine and H_2_O_2_. This effect was associated with a significant increase in H_2_O_2_-induced Bax expression and activation of caspase-3 and PARP in C6 cells. The authors concluded that methylguanidine could contribute to neurodegeneration associated with uremia by enhancing the pro-apoptotic effect of H_2_O_2_ and via alteration of mitochondrial calcium homeostasis in glial cells. Further studies are needed to evaluate whether the effect of methylguanidine on oxidative stress-induced astrocyte apoptosis is involved in CKD-related dementia. Recent evidence suggests that oxidative stress also compromises neurological recovery after stroke by causing glial cell death [19]. In future studies, it would be interesting to evaluate whether the oxidative stress-induced astrocyte apoptosis that occurs in response to methylguanidine alters post-stroke recovery in CKD.

#### 4.2.2. Indoxyl Sulfate

The inflammatory reaction that occurs after stroke is known to progressively induce astrogliosis [172,173]. These reactive astrocytes then release growth-inhibitory molecules that chemically prevent axonal extensions and secrete ROS, pro-inflammatory cytokines and matrix metalloproteinases that accentuate ischemic stroke damage. In a recent study, IS was reported to promote iNOS and cyclooxygenase-2 (COX-2) expression, together with TNF-α and IL-6 release and nitrotyrosine formation in primary mouse astrocytes and mixed glial cells [174]. The same group reported also that IS decreased the expression of superoxide dismutase (SOD), an enzyme known for its antioxidant properties, in the C6 glioma cell line cultured in vitro, an effect associated with increased ROS production [174]. Altogether, these data suggest that IS-induced oxidative stress and subsequent astrogliosis generate a neurotoxic environment. Further studies will be needed to investigate the impact of IS-induced glial cell activation and subsequent inflammation on the stroke damage and neurodegeneration observed in CKD [174].

#### 4.2.3. Quinolinic Acid

Quinolinic acid has been reported to increase IL-1β production in human astrocytes and concomitantly induce a marked increase in glial fibrillary acid protein levels and a reduction of vimentin levels, features consistent with astrogliosis [135]. Quinolinic acid also induces astrocytes to produce large quantities of MCP-1 (CCL2), RANTES (CCL5), and IL-8 (CXCL8) and upregulate the expression of chemokine receptors CXCR4, CCR5, and CCR3 [175]. Most of these effects are comparable to those induced by the classical mediators of inflammation, such as TNF-α, IL-1β, and IFN-γ [175]. These results suggest that this UT might be critical in the amplification of brain inflammation in CKD.

### 4.3. Neurons

Cognitive dysfunction is associated with a neuronal apoptosis response. Similarly, ischemic stroke triggers complex cellular events that progressively lead to both apoptotic and necrotic neuronal cell death. Oxidative stress is involved in nearly all neurological disorders [176] and several studies have suggested that accumulation of ROS results in neuronal damage and triggers apoptosis [177]. Uremic toxin-induced oxidative stress has been reported to promote cell death and cell senescence in various cell types [55,178,179,180,181]. UTs may, therefore, also promote cognitive dysfunction or impair post-stroke recovery via a direct neurotoxic action induced by increased oxidative stress. In line with this hypothesis, increased neuronal damage has been observed in mice following IS injection, a phenomenon associated with increased COX-2 expression and nitrotyrosine formation in brain tissue [174].

#### 4.3.1. Guanidino Compounds

Similarly, guanidino compounds have been reported to exert a direct neurotoxic effect [26] by blocking GABA-A and activating glutamate *N*-methyl-*d*-aspartate (NMDA) receptors, thereby increasing post-synaptic calcium levels and calcium-triggered events [182,183].

#### 4.3.2. Homocysteine

Apart from its previously discussed role on coagulation and endothelial dysfunction, hyperhomocysteinemia could also impair neuronal pathways and display direct neurotoxic effects. Indeed, in cultures of cortical neurons, homocysteine caused direct neurotoxicity by activating the NMDA subtype of glutamate receptor [184]. Excessive stimulation of these receptors is known to mediate brain damage in focal ischemia [185,186]. Thus, homocysteine may not only be associated with the vascular injury leading to stroke, but may also participate in the ensuing neurotoxic response in the brain. As discussed previously, a prospective cohort study reported that plasma homocysteine was an independent risk factor for dementia [90]. In this study, higher plasma homocysteine levels were associated with smaller brain volume and the presence of silent brain infarcts, even in healthy, middle-aged adults. Clinical studies have also shown that elevated plasma homocysteine concentrations are associated with an increased risk of Alzheimer's disease [91]. Whether the cognitive disorders observed in response to hyperhomocysteinema appear as a consequence of NDMA receptors activation needs to be elucidated. Further studies are needed to evaluate the impact of hyperhomocysteinemia in the neurological damage associated with CKD.

#### 4.3.3. β-2-Microglobulin

β-2-microglobulin accumulation in blood with aging has been reported to promote age-related cognitive dysfunction and impaired neurogenesis [187], a key phenomenon allowing brain parenchyma regeneration [188]. Neurogenesis is primordial to promote stroke recovery [189,190] and neurogenesis deficiency is considered to be a major cause of cognitive impairment [191]. Therefore, the possibility that B2M-induced inhibition of neurogenesis may account for CKD-induced poor stroke recovery cannot be ruled out and should be investigated.

#### 4.3.4. Lanthionine

Cystathionine γ-lyase appears to be the predominant enzymatic source of H_2_S in the vasculature and heart [39], whereas in the central nervous system CBS predominates [39,41,192]. Since CBS is expressed and produces H_2_S in the brain and because the endogenous concentration of H_2_S in the brain is relatively high, it has been suggested that H_2_S may play a role in synaptic transmission [41]. Interestingly, physiological concentrations of H_2_S selectively enhance NMDA receptor-mediated responses and facilitate the induction of hippocampal long-term potentiation, suggesting that H_2_S functions as a neuromodulator involved in associative learning [193]. In addition, a growing body of evidence has shown that H_2_S mediates signals between neuronal cells and astrocytes by increasing Ca^2+^-influx in order to maintain calcium homeostasis and regulate synaptic activity [193,194]. Previous studies have indicated that H_2_S is involved in the pathophysiological process of ischemia-reperfusion injury and shock, and exerts protective effects on neurons. Indeed, H_2_S was reported to prevent oxygen-glucose deprivation/reoxygenation-induced apoptosis via improving mitochondrial dysfunction and suppressing an ROS-mediated caspase-3 pathway in mouse cortical neurons [195]. In other studies, H_2_S was reported to offer neuroprotection against traumatic brain injury in rats [196] and mice [197,198] through decreased oxidative stress and reduced apoptosis. Exogenous H_2_S administration in rats also protected against global cerebral ischemia/reperfusion injury via its anti-oxidative, anti-inflammatory and anti-apoptotic effects in rats [199]. These data suggest that lanthionine-induced impaired H_2_S production may play a central role in the onset and worsening of CKD-related cerebrovascular diseases. Further studies will be needed to clarify this concern.

## 5. Conclusions

**Figure 2:**
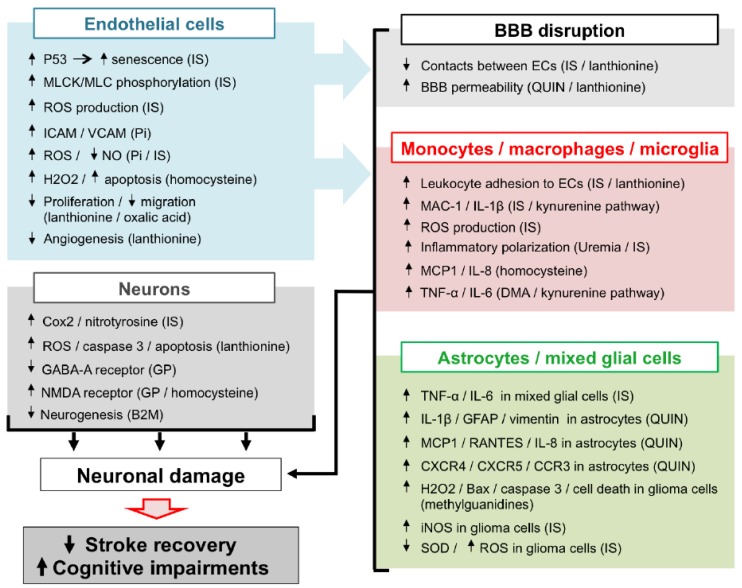
Impact of uremic toxins on neurological damage: a mechanistic view based on a review of the recent literature. B2M: β-2-microglobulin, BBB: blood-brain barrier, DMA: dimethylarginines, ECs: endothelial cells, GFAP: glial fibrillary acidic protein, GP: guanidino compounds, iNOS: inducible nitric oxide synthase, IS: indoxyl sulfate, MLCK: myosin light chain kinase, MLC: myosin light chain, NO: nitric oxide, Pi: inorganic phosphate, QUIN: quinolinic acid, ROS: reactive oxygen species, SOD: superoxide dismutase. ICAM: intercellular adhesion molecule, VCAM: vascular cell adhesion molecule, GABA-A: γ-aminobutyric acid receptor A, NMDA: *n*-methyl-*d*-aspartic acid, MCP1: Monocyte chemoattractant protein 1, MAC-1: macrophage-1 antigen, TNF-α: tumour necrosis factor, CXCR4: C-X-C chemokine receptor type 4, CXCR5: C-X-C chemokine receptor type 5, CCR3: C-C chemokine receptor type 3, RANTES: Regulated on Activation, Normal T Expressed and Secreted

In CKD patients, the accumulation of UTs is responsible for peripheral vascular dysfunction (endothelial dysfunction, atherosclerosis, vascular calcification, hypertension due to overactivation of RVLM neurons), which alters the general hemodynamic and favours the occurrence of cerebrovascular diseases. In addition, evidence from in vitro studies and in vivo animal experiments suggests that UTs may display direct deleterious effect in the brain microenvironment (Figure 2). Among the main mechanisms involved in these local effects, the endothelial dysfunction induced by molecules, such as Pi and IS may lead to BBB disruption and leukocyte adhesion, resulting in increased leukocyte infiltration into the damaged brain. Subsequent exposure of both macrophages and astrocytes to UTs amplifies the release of inflammatory cytokines and oxidative stress in the brain microenvironment. These deleterious mechanisms, together with the direct neurotoxic properties of certain UTs, promote neuronal death. Altogether, these mechanisms may account for the accelerated cognitive decline and poor stroke outcomes observed in CKD patients. However, to the best of our knowledge, evidence on the existence of a causal link between increased neurological disorders and UT-induced brain damage is still lacking. It should be noted that no animal models are currently available to study stroke and cognitive impairment in CKD. Such models need to be developed in order to advance research in this field. This review highlights the fact that IS is currently the most extensively studied UT in the context of cerebral disorders. In particular, IS is the only UT that has been reported to promote endothelial cell dysfunction, oxidative stress and inflammation and to induce glial cell activation and neuronal death. IS has also been reported to induce senescence of both vascular [99] and tubular cells [200] in the CKD setting. Cerebral cell senescence could, therefore, occur as a result of IS accumulation in the brain. In addition, uremic toxins such as IS are known to participate in the pathogenesis of stroke by amplifying atherosclerosis [46,201] and hypertension [29]. In vitro evidence suggests that reduction of IS by AST-120 attenuates monocyte inflammation [123], oxidative stress and endothelial dysfunction [202,203] related to CKD. Targeting IS, therefore, appears to be a promising strategy to protect CKD patients from brain damage. Interestingly, in a retrospective analysis, 3 to 5 years of treatment with AST-120 decreased the prevalence of stroke events in pre-dialysis CKD patients [204]. However, interventional studies designed to evaluate the impact of AST-120 on stroke outcomes in animals or patients with CKD are lacking and, therefore, need to be conducted. In the future, it would also be interesting to evaluate the impact of AST-120 administration in CKD animals presenting various neurological disorders, such as stroke or cognitive impairment. As discussed throughout this review, oxidative stress plays a central role in IS-induced endothelial cell dysfunction, inflammation, astrocyte activation, and neuronal death and is also a key mediator in the effects of other UTs. Studies on the impact of inhibition of pro-oxidative enzymes, such as NADPH oxidase [205], or myeloperoxidase [18], or increased antioxidant enzyme expression [206] in uremic animals or CKD patients should, therefore, also be envisaged.
